# Supplementary material for: Comprehensive micro-scaled proteome and phosphoproteome characterization of archived retrospective cancer repositories
Source: Nat Commun. 2021 Jun 11;12:3576. doi: 10.1038/s41467-021-23855-w (PMC8196151; doi:10.1038/s41467-021-23855-w)
Supplement: Supplementary file 1 — Supplementary Information [file 41467_2021_23855_MOESM1_ESM.pdf]

Supplementary Information

Comprehensive micro-scaled proteome and phosphoproteome characterization of archived retrospective cancer repositories

Corinna Friedrich[1,2,3,4,5], Simon Schallenberg[3], Marieluise Kirchner[6,7], Matthias Ziehm[6,7], Sylvia Niquet[6,7], Mohamed Haji[6], Christin Beier[6], Jens Neudecker[8], Frederick Klauschen*[1,2,3,7,9], Philipp Mertins*[1,2,6,7]

[1]German Cancer Consortium (DKTK), partner site Berlin

[2]German Cancer Research Center (DKFZ), Heidelberg, Germany

[3]Institute of Pathology, Charité - Universitätsmedizin Berlin, corporate member of Freie Universität Berlin, Humboldt-Universität zu Berlin, and Berlin Institute of Health, Berlin, Germany

[4]Max Delbrück Center for Molecular Medicine in the Helmholtz Association (MDC), MDC graduate school, Berlin, Germany

[5]Humboldt Universität zu Berlin, Institute of Chemistry, Berlin, Germany

[6]Max-Delbrück-Center for Molecular Medicine in the Helmholtz Association (MDC), Proteomics Platform, Berlin, Germany

[7]Berlin Institute of Health at Charité – Universitätsmedizin Berlin, Berlin, Germany

[8]Department of Surgery - Campus Charité Mitte and Campus Virchow-Klinikum, Charité - Universitätsmedizin Berlin, Berlin, Germany

[9]Institute of Pathology, Ludwig-Maximilians-Universität München, Munich, Germany

*corresponding authors, email: frederick.klauschen@charite.de or philipp.mertins@mdc-berlin.de

Supplementary Fig. 1

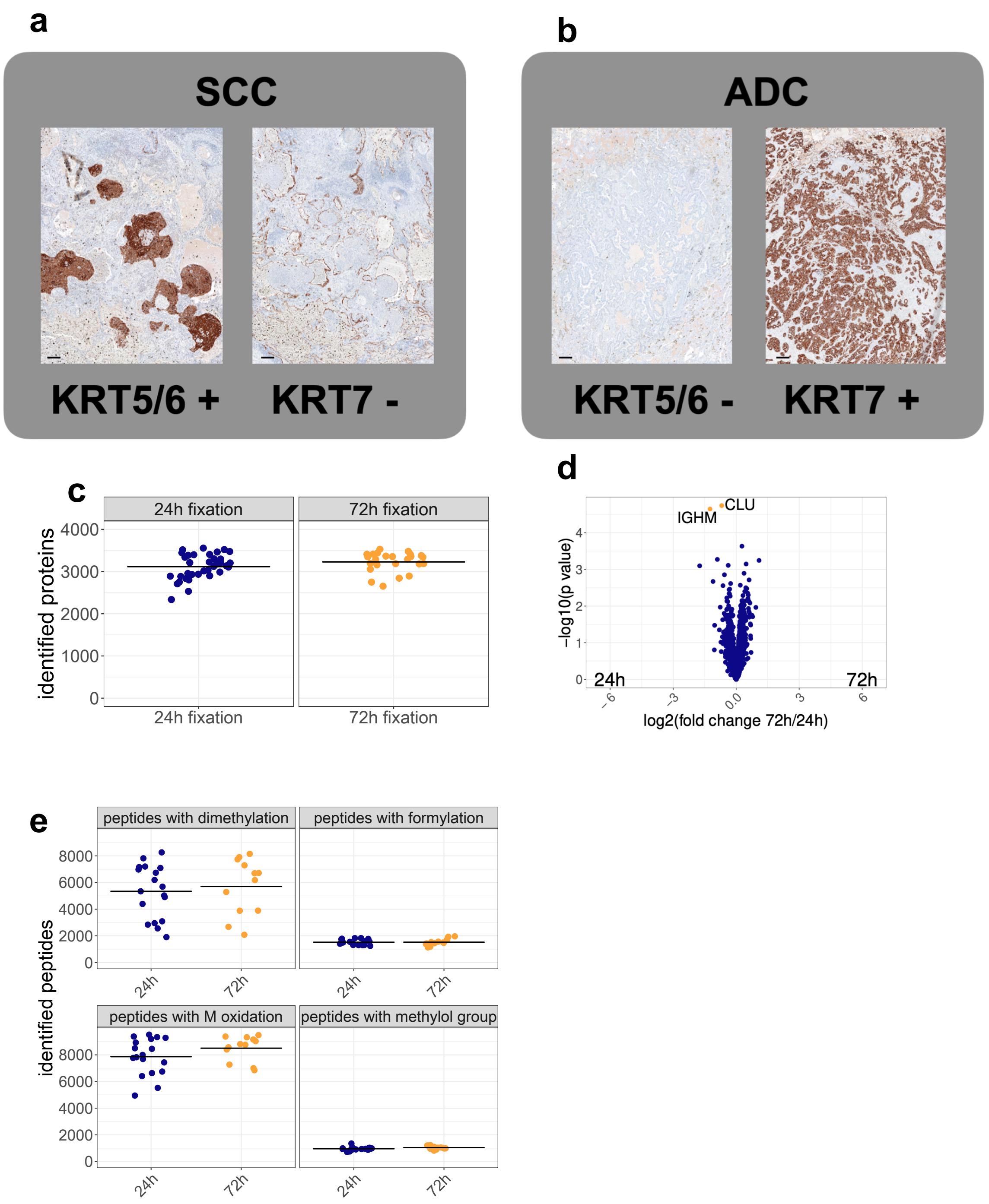

Supplementary figure 1: Influence of fixation time on protein identification.

a: Lung squamous cell carcinoma (SCC) slice with routine immunohistochemical (IHC) staining (representative result) for cytokeratin (KRT) 5/6 (left) and KRT7 (right). Cytokeratin 5/6 only present in the tumor cells is stained dark brown. The black scale bar at the bottom left depicts 0.2 mm.

b: Lung adenocarcinoma (ADC) with routine IHC staining (representative result) for KRT5/6 (negative, left) and KRT7 (positive, right). KRT7 which is only expressed in the secretory tumor cells is stained dark brown. The black scale bar depicts 0.2 mm.

c: Identified proteins in 30 FFPE lung cancer tissues that were fixed either 24h or 72h, the black bar is showing the mean of the group (mean peptides after 24h = 28,173, mean peptides after 72h = 29,709, two-sided t-test p value = 0.15). Source data are provided as a Source Data file.

d: Volcano plot depicting the label-free comparison of FFPE samples fixed in formalin for 24h or 72h. Significantly regulated proteins (two-sided moderated t-test, Benjamini-Hochberg-adjusted p<0.05, i.e. 5% FDR) are shown in orange and their gene names are annotated. Source data are provided as a Source Data file.

e: Identified peptides with demethylation, formylation, methionine oxidation or an added methylol group in 30 FFPE lung cancer tissues depending on fixation time, the black bar is showing the mean of the group (mean modified peptides 24h vs 72h: demethylation: 5,343 vs 5,711, two sided t-test p-value 0.64, formylation: 1,523 vs 1,526, two sided t-test p-value = 0.97, M oxidation: 7.864 vs 8,501, two sided t-test p-value = 0.09, methylol group: 955 vs 1,039, two sided t-test p-value = 0.14). Source data are provided as a Source Data file.

Supplementary Fig. 2

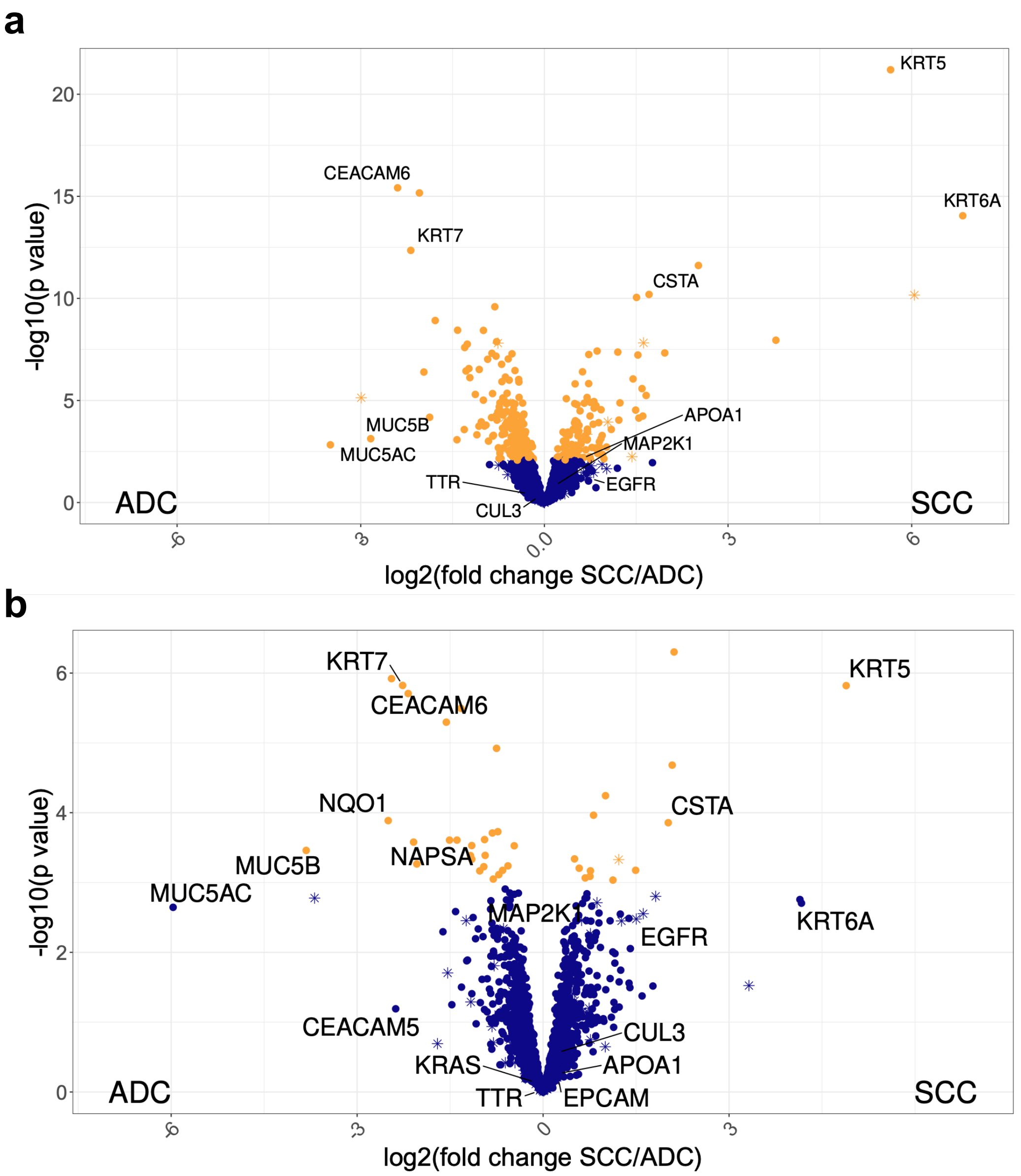

Supplementary figure 2: Label-free proteome analysis allows us to distinguish abundant ADC- and SCC-specific marker proteins in a reduced sample set.

a: Label-free comparison of 16 ADC and 14 SCC FFPE samples with a two-sided moderated t-test. Significantly regulated proteins (Benjamini-Hochberg-adjusted p<0.05, i.e. 5% FDR) are shown in orange and NSCLC-relevant marker proteins are annotated. Proteins that fall outside of a 95% prediction interval in the scatterplot comparing ADC and SCC replicates (Supplementary Fig. 3B/3C) are shown with an asterisk symbol. Source data are provided as a Source Data file.

b: Label-free comparison of a subset of 5 ADC and 5 SCC FFPE samples that were later on used for TMT experiments with a two-sided moderated t-test. Significantly regulated proteins (Benjamini-Hochberg-adjusted p<0.05, i.e. 5% FDR) are shown in orange and NSCLC-relevant marker proteins are annotated. Proteins that fall outside of a 95% prediction interval in the scatterplot comparing ADC and SCC replicates (Supplementary Fig. 3B/3C) are shown with an asterisk symbol. Source data are provided as a Source Data file.

Supplementary Fig. 3

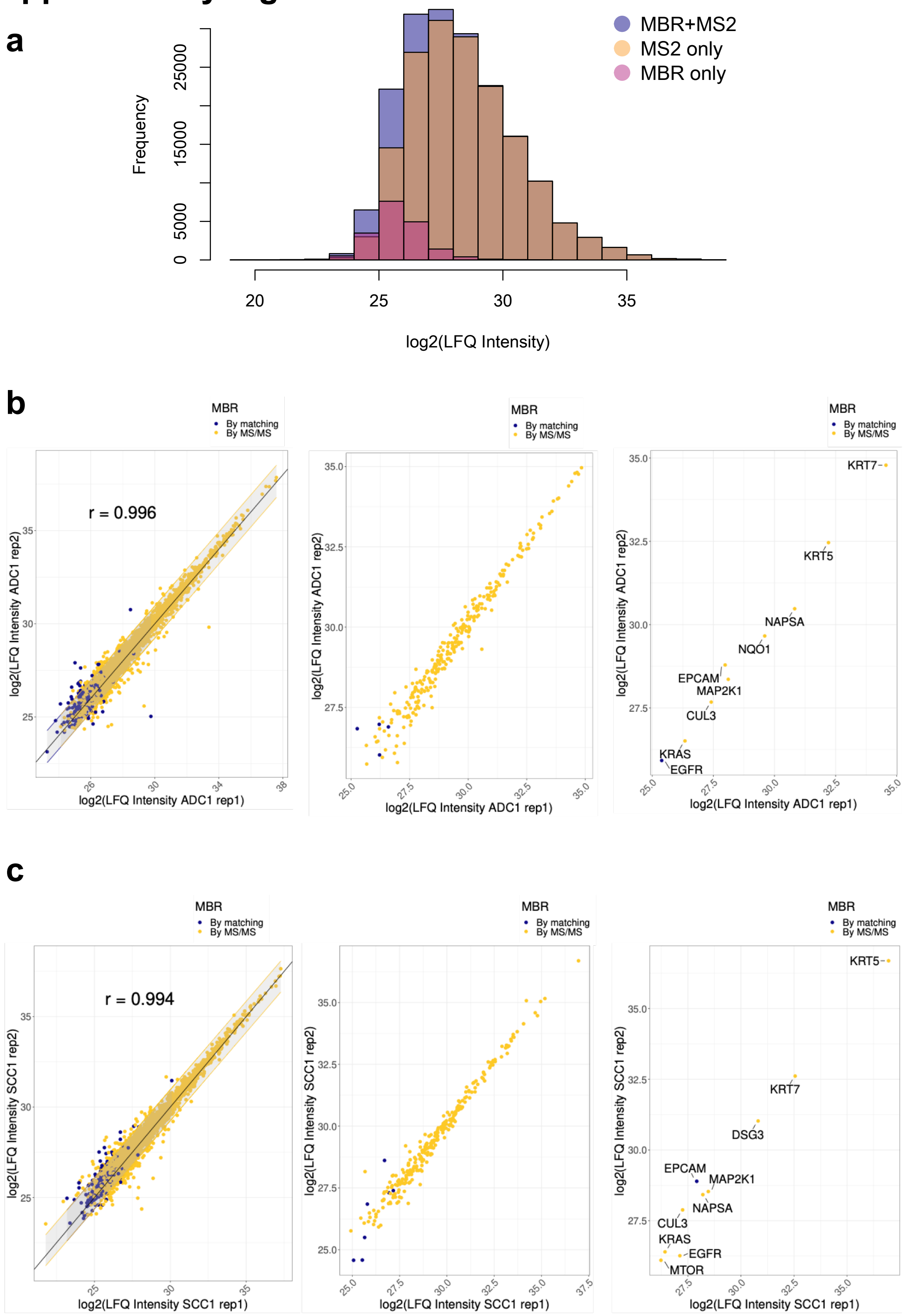

Supplementary figure 3: Small contribution of the match-between-runs (MBR) algorithm in MaxQuant to the number of quantified proteins in the LFQ experiment.
a: Histogram showing the LFQ intensity distribution of all proteins (blue), those identified from MS/MS spectra (orange) and only identified with the MBR algorithm (magenta).
b: Comparison of LFQ intensities in two ADC replicates for all quantified proteins (left), only significantly regulated proteins (middle) and for NSCLC-relevant marker proteins (right). Proteins identified by MS/MS spectra shown in yellow, proteins identified via MBR algorithm in blue. The Pearson correlation coefficient for the technical replicate pairs is 0.996 ($p<0.01$) and the average Pearson correlation coefficient across ADC samples is 0.888. The

comparison of the intensity of all proteins includes 95% prediction intervals in light grey. Source data are provided as a Source Data file.

c: Comparison of LFQ intensities in two SCC replicates for all quantified proteins (left), only significantly regulated proteins (middle) and for NSCLC-relevant marker proteins (right). Proteins identified by MS/MS spectra shown in yellow, proteins identified via MBR algorithm in blue. The Pearson correlation coefficient for the technical replicate pairs is 0.994 (p<0.01) and the average Pearson correlation coefficient across SCC samples is 0.917. The comparison of the intensity of all proteins includes 95% prediction intervals in light grey. Source data are provided as a Source Data file.

Supplementary Fig. 4

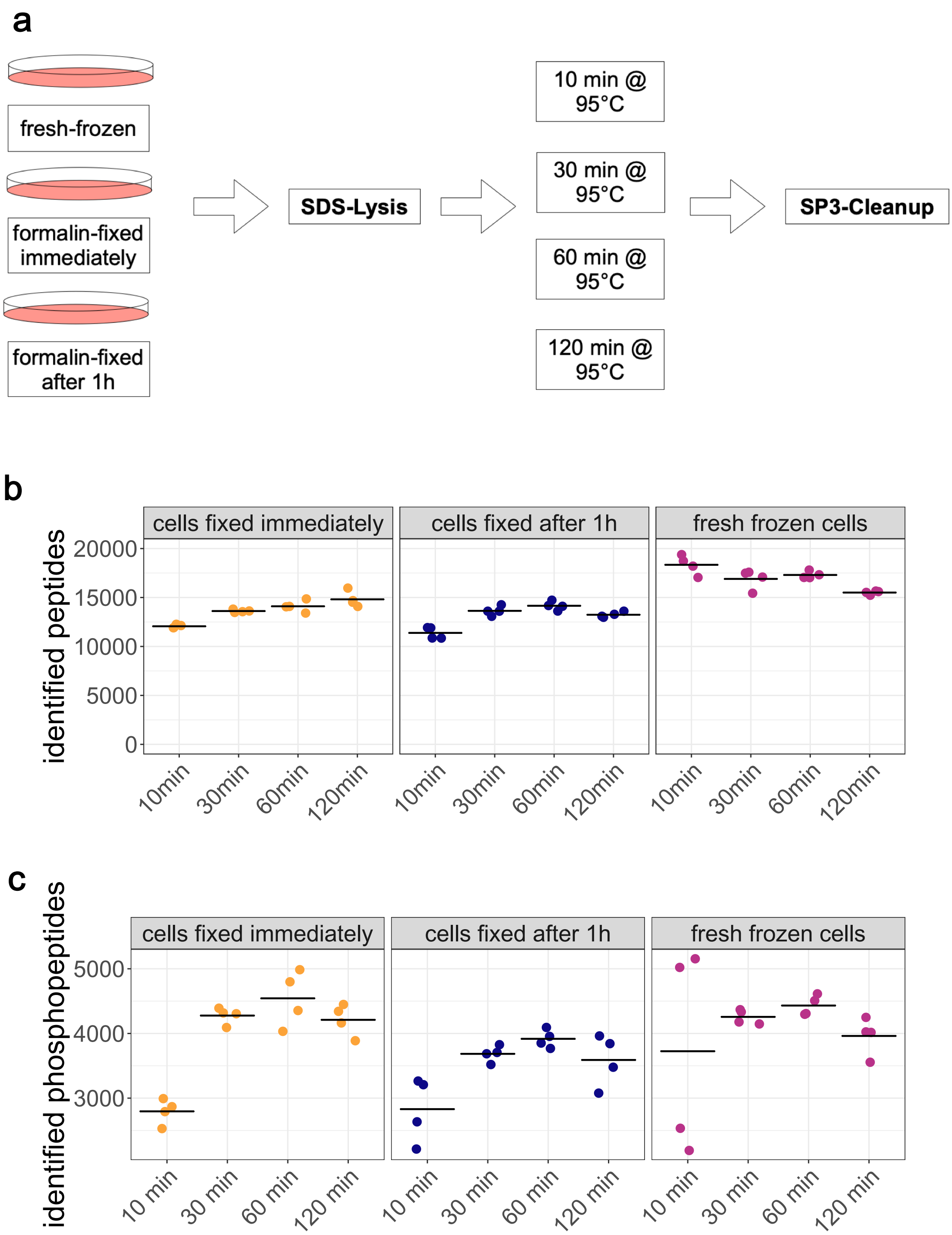

Supplementary figure 4: Prolonged lysis at 95°C is beneficial for FFPE proteome and to some extend FFPE phosphoproteome coverage.

a: HEK cells that were either frozen directly after harvesting, formalin-fixed immediately on the plate before harvesting or first incubated for 1h at 4°C, then formalin-fixed and harvested were lysed and aliquots were incubated at 95°C for either 10 min, 30 min, 60 min or 120 min before SP3 clean-up.
b: Identified peptides in the three types of samples after 10 min, 30 min, 60 min, and 120 min of lysis at 95°C. The black bar shows the mean of the group (n = 4 biological replicates). Source data are provided as a Source Data file.
c: Identified phosphopeptides in fresh-frozen, immediately fixed cells and cells fixed after 1h over the four time points. 100 µg peptide from each sample had been enriched for phosphopeptides with automated IMAC. The black bar shows the mean of the group (n = 4 biological replicates). Source data are provided as a Source Data file.

Supplementary Fig. 5

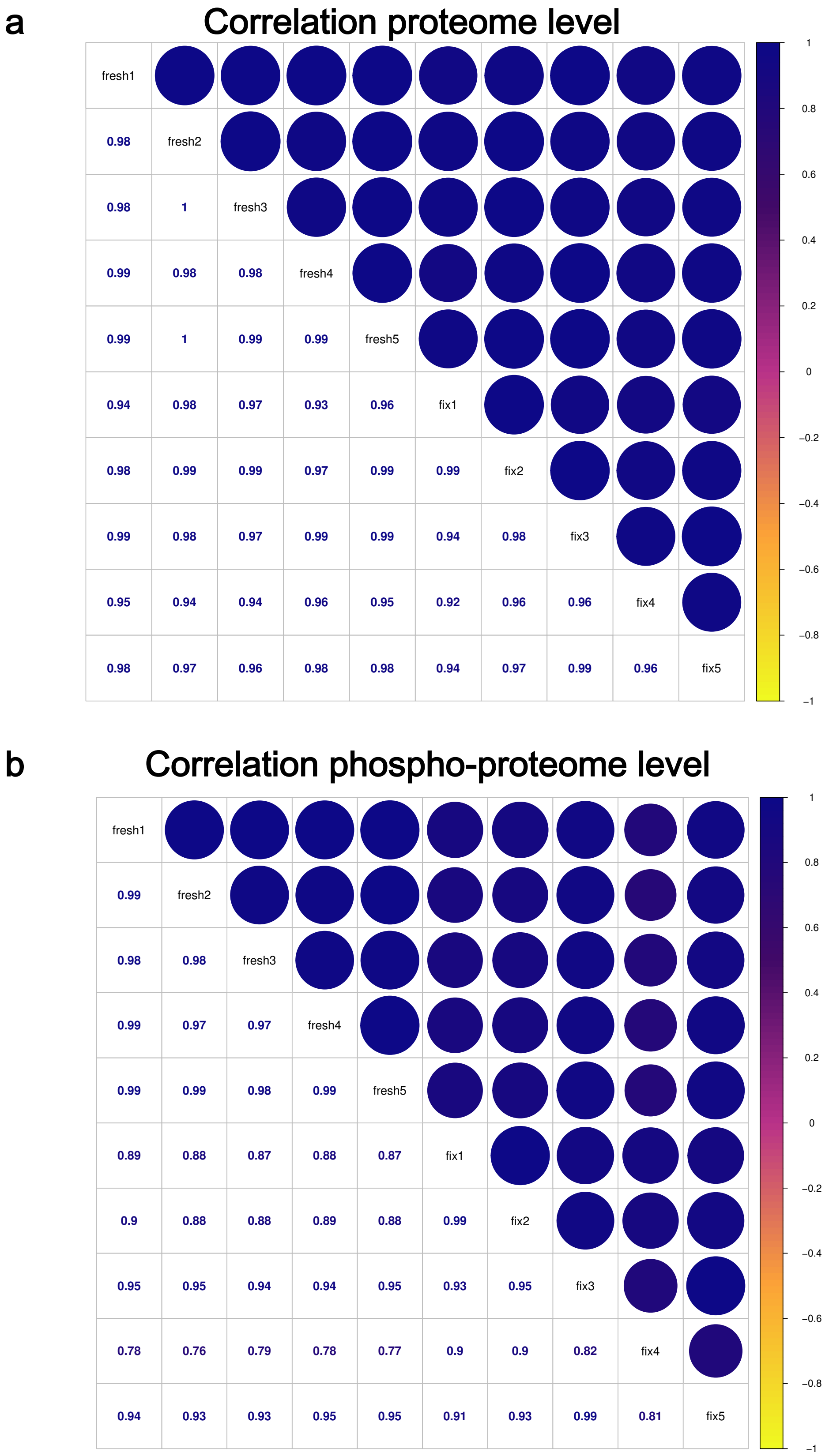

Supp. Fig. 5: Strong correlation of the proteomes and phosphoproteomes of fresh-frozen and formalin-fixed HEK293 cells.
a: Correlation of TMT reporter ion protein intensities for fresh-frozen and formalin-fixed HEK293 cells shown in a correlation matrix with pearson correlation coefficients of 0.96-1 ($p< 0.01$). Strong correlations are shown in dark blue.

b: Correlation matrix showing the pearson correlation coefficients (0.7-0.97, p<0.01) of the TMT reporter ion intensities on the phosphoproteome level for fresh-frozen and formalin-fixed HEK293 cells. Strong correlations are shown in dark blue.

Supplementary Fig. 6

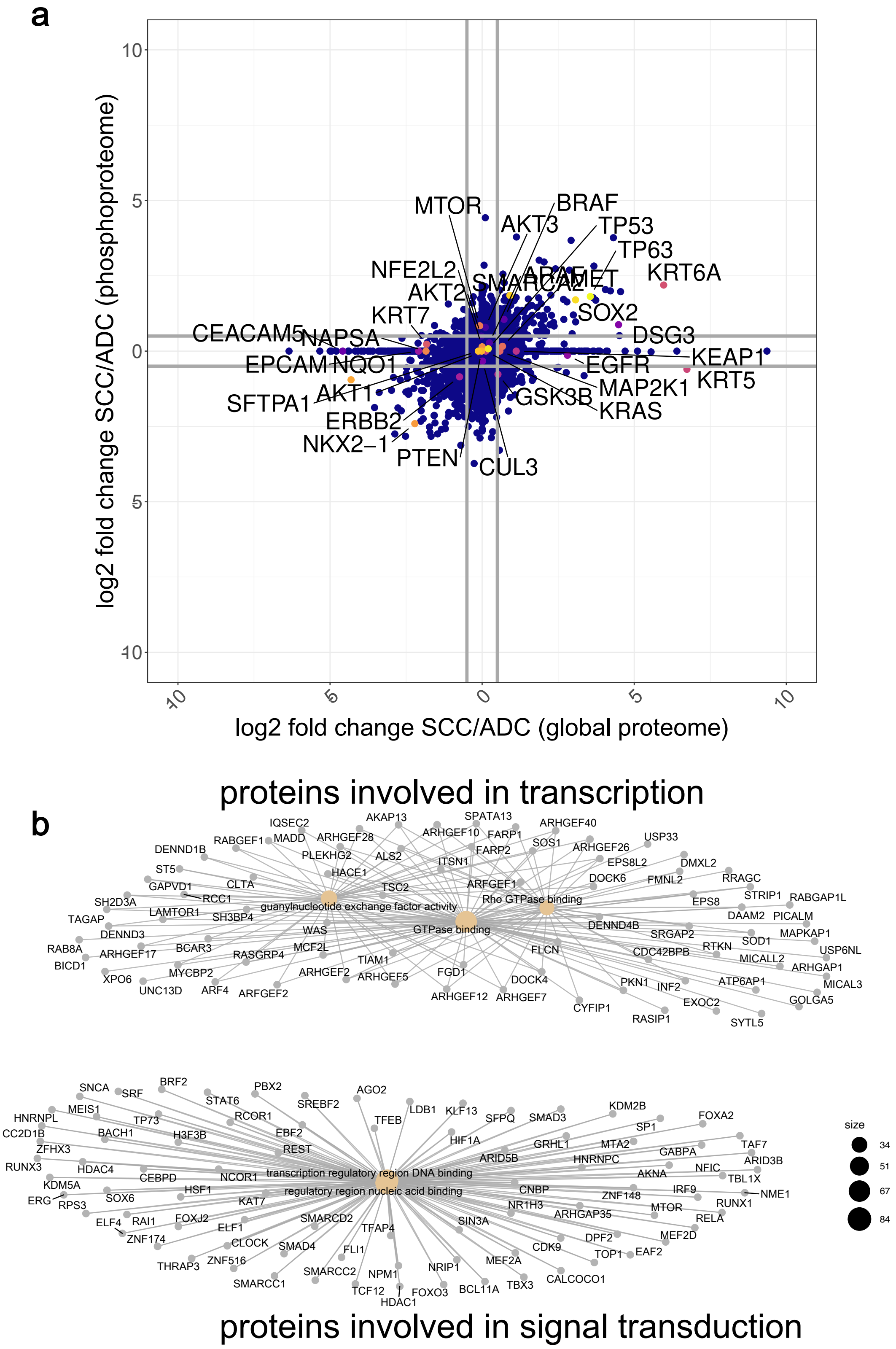

Supplementary figure 6: TMT equal loading experiments reveal ADC- and SCC-specific proteins that were found to be differential by proteome, phosphoproteome profiling, or both.

a: Comparison between log2 fold change ratios of ADC/SCC for global proteome and phosphoproteome in equal loading TMT. Intersecting vertical and horizontal lines at log2 fold change -0.5 and 0.5 show which genes are only changed on a global or phosphoproteome level. Proteins (X-axis) and phosphosites (Y-axis) that directly lie on the axis were only detected in one data type. NSCLC-relevant proteins are highlighted. Source data are provided as a Source Data file.
b: GO molecular function analysis of proteins only regulated on a phosphoproteome level and not on global proteome level for equal loading TMT.

Supplementary Fig. 7

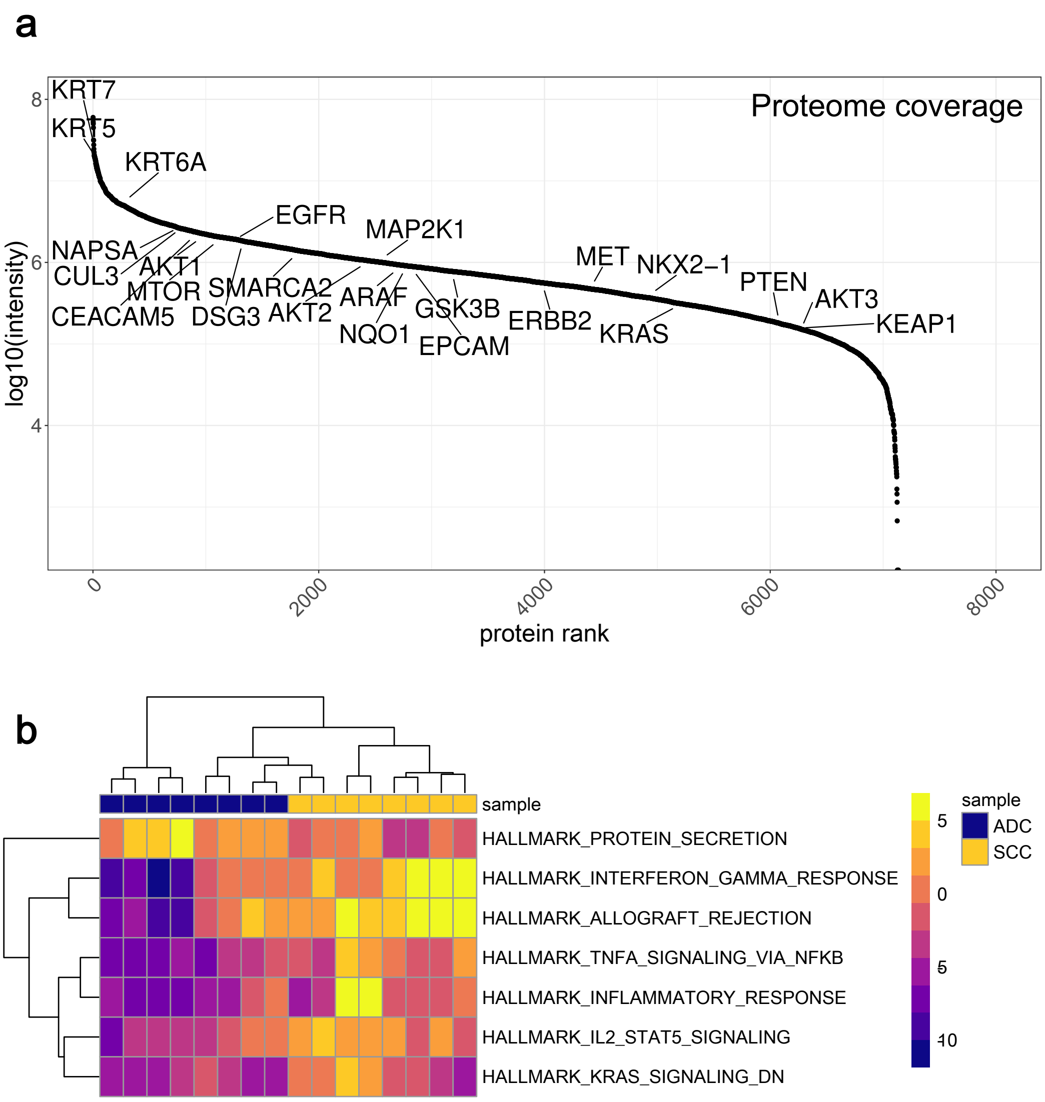

Supplementary figure 7: Coverage and hallmarks of cancer pathways observed for microscaled TMT experiments.
a: Log10 reporter ion intensity distribution over all proteins that were quantified in microscaled TMT experiments. Lung cancer-relevant gene names are indicated.
b: Heatmap showing the 7 hallmarks of cancer gene sets that were significantly upregulated when comparing the results of a ssGSEA hallmarks of cancer analysis between ADC and SCC. High enrichment scores are shown in yellow, low enrichment scores in dark blue.

Supplementary Fig. 8

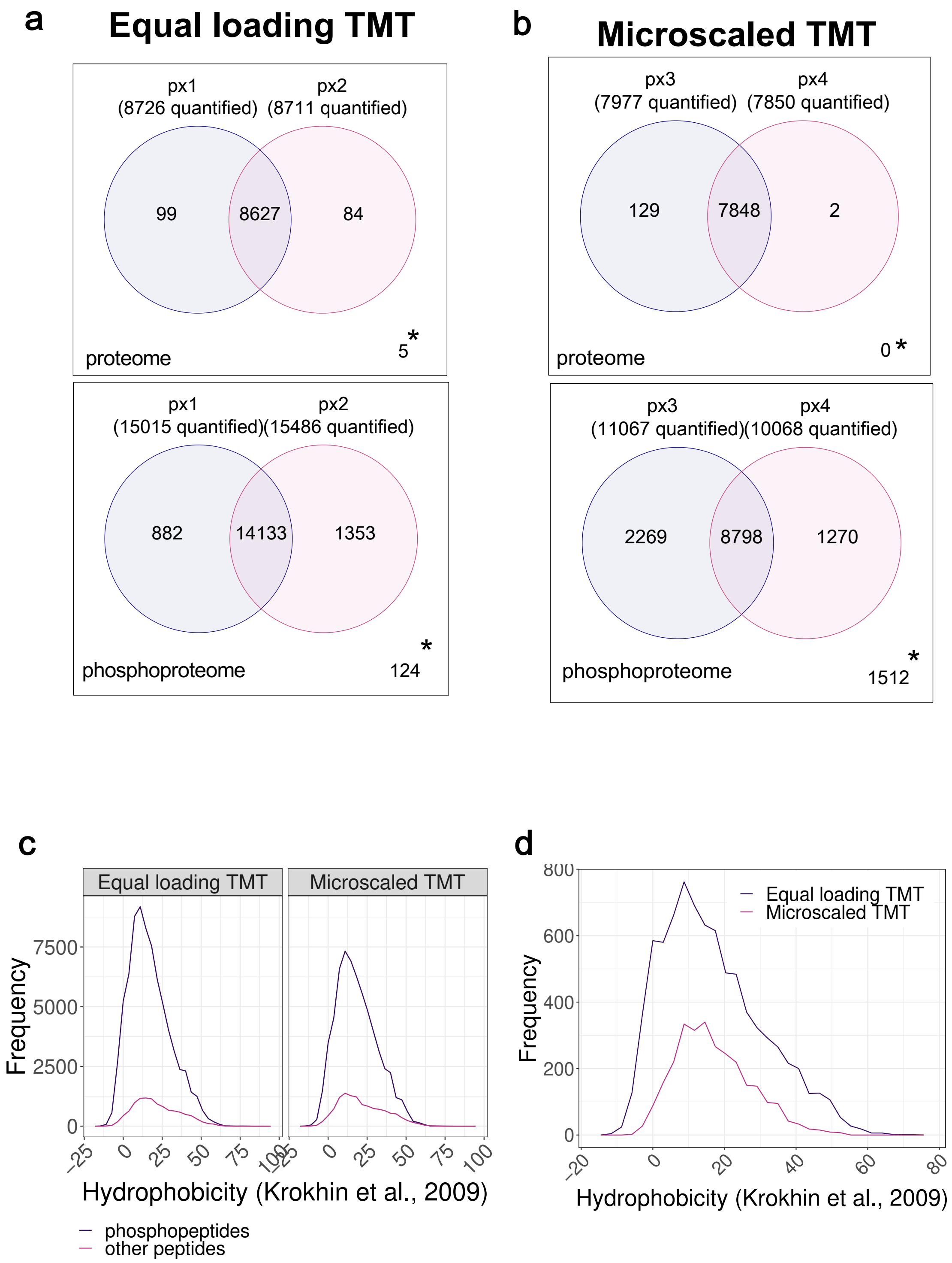

Supplementary figure 8: Variability between equal loading TMT and microscaled TMT.
a: Venn diagrams depicting the overlap between two replicate TMT plexes in equal loading TMT on proteome level (top) and phosphoproteome level (bottom). *marks proteins/phosphosites identified but not quantified in either plex (5 proteins and 124 phosphosites).
b: Venn diagrams depicting the overlap between two replicate TMT plexes in microscaled TMT on proteome level (top) and phosphoproteome level (bottom). *marks proteins/phosphosites identified but not quantified in either plex (0 proteins and 1512 phosphosites).
c: Frequency distribution of phosphopeptides and all other peptides over the hydrophobicity index [1] for equal loading TMT and microscaled TMT. Source data are provided as a Source Data file.

d: Frequency distribution of phosphopeptides only quantified in equal loading TMT (blue) and microscaled TMT (magenta) over the hydrophobicity index[1]. Source data are provided as a Source Data file.

Supplementary Fig. 9

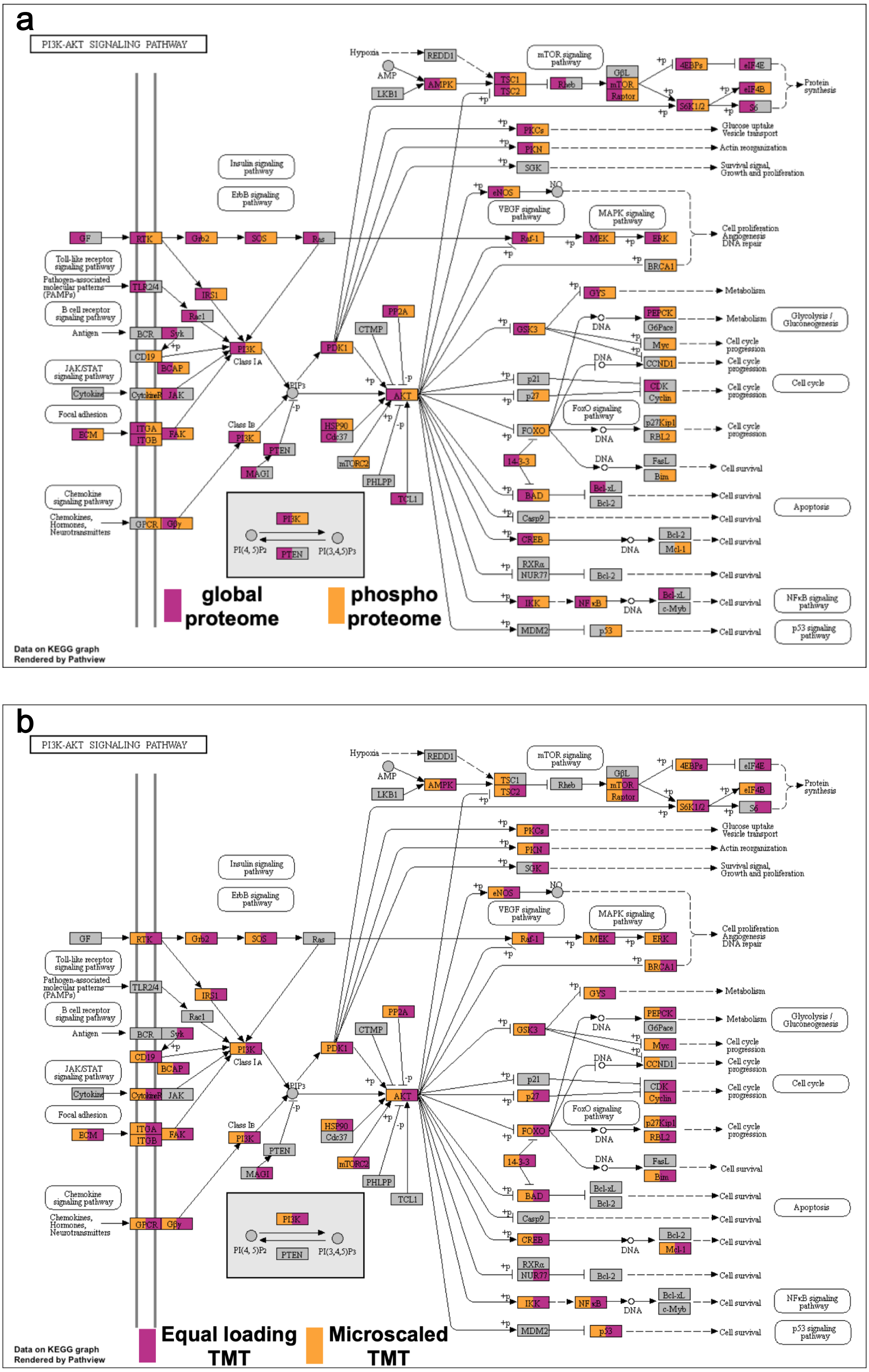

Supplementary figure 9: PI3K-Akt signaling pathway coverage in microscaled TMT experiments.

a: PI3K-Akt signaling pathway coverage on microscaled TMT experiments on a global proteome (magenta) and phosphoproteome (orange) level.
b: PI3K-Akt signaling pathway coverage on a phosphoproteome level by equal loading TMT (magenta) and microscaled TMT (orange) experiments.

Supplementary Fig. 10

a

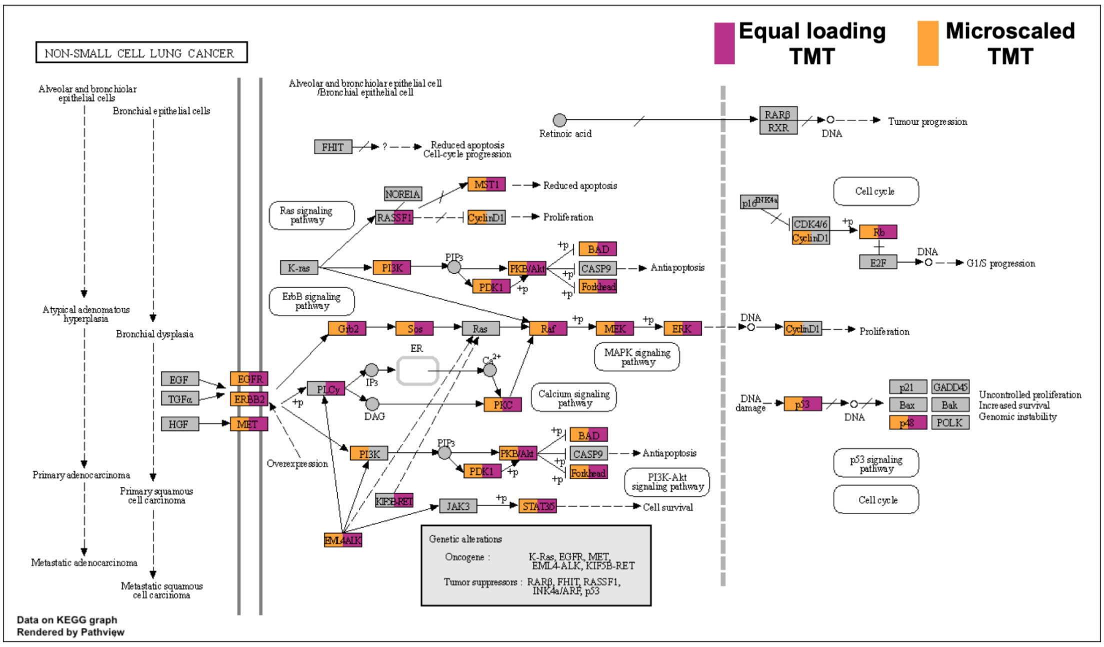

b

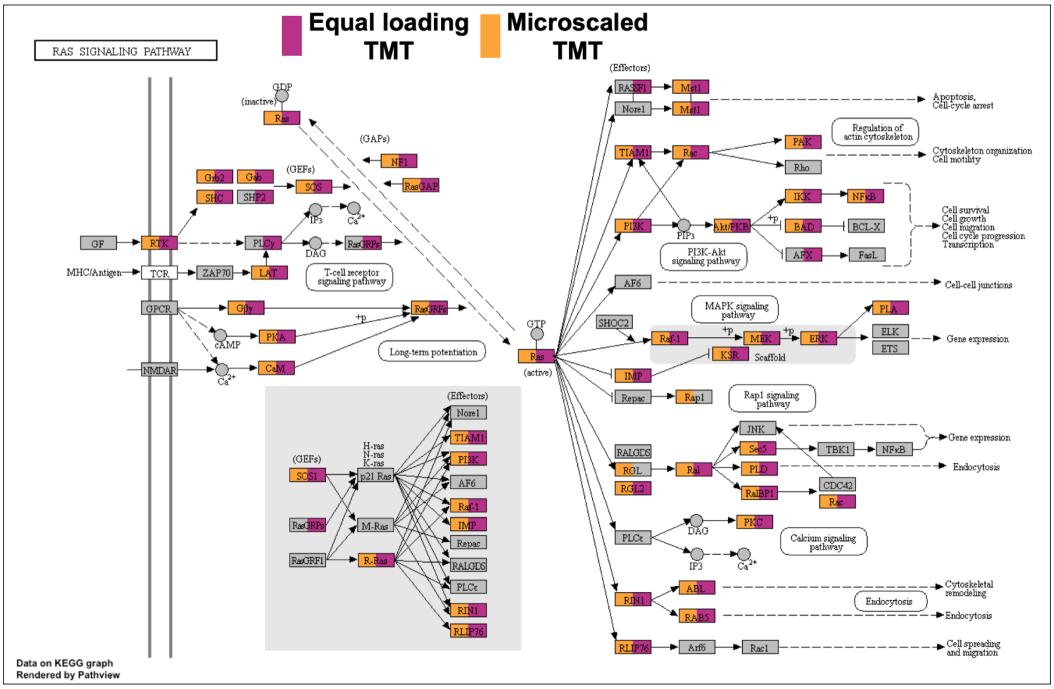

Supplementary figure 10: Signaling pathway coverage on a phosphopeptide level in equal loading and microscaled TMT experiments.

a: Non-Small cell lung cancer pathway coverage on phosphoproteome level in equal loading TMT (magenta) and microscaled TMT (orange).
b: RAS signaling pathway coverage on phosphoproteome level in equal loading TMT (magenta) and microscaled TMT (orange).

Supplementary Fig. 11

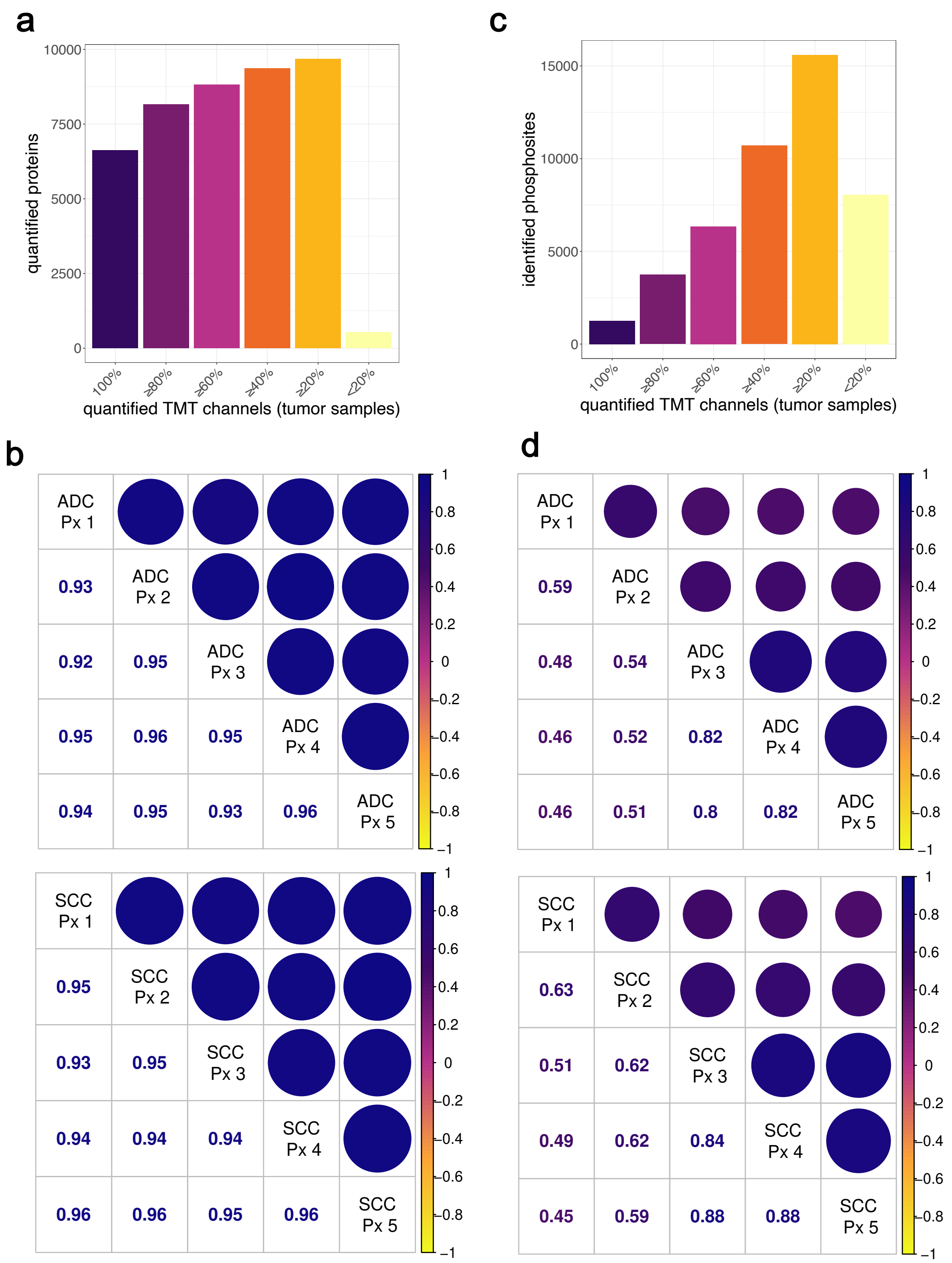

Supplementary figure 11: Identification and quantification in microscaled TMT over multiple replicate plexes.
a: Barplot showing the number of quantified TMT channels per tumor sample on the proteome level. Source data are provided as a Source Data file.
b: Pearson correlation matrices of mean protein reporter ion intensities per plex for ADC (top) and SCC (bottom). Strong correlations are shown in dark blue.
c: Barplot showing the number of quantified TMT channels per tumor sample on the phosphoproteome level. Source data are provided as a Source Data file.

d: Pearson correlation matrices of mean phosphosite reporter ion intensities per plex for ADC (top) and SCC (bottom). Strong correlations are shown in dark blue.

Supplementary Fig. 12

a

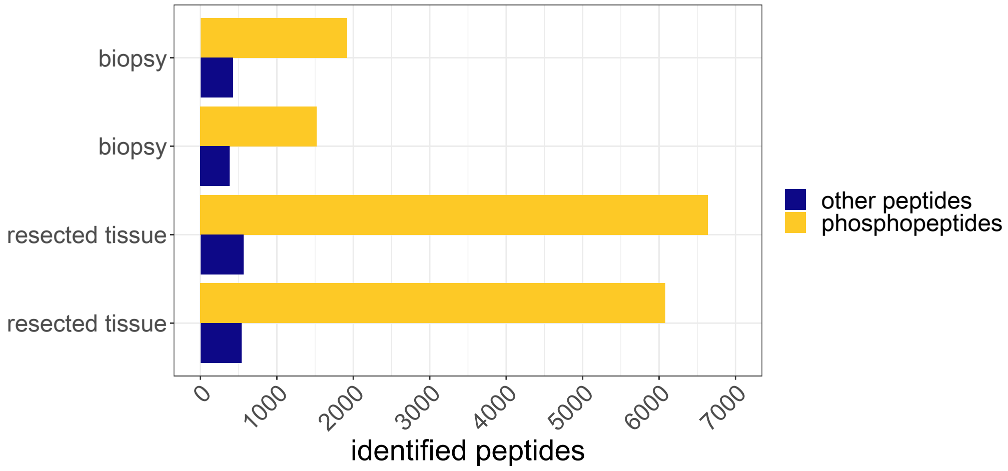

b

		approx. protein yield per 1x10 µm slice	identified proteins per 1 µg injection (LFQ)	identified phosphosites from 100 µg peptide (LFQ)	quantified proteins in TMT11 experiment	quantified phosphosites in TMT11 experiment
**Resected tissue**	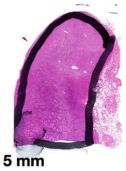 5 mm	139 µg +/- 104 µg	4428 +/- 290	6361 +/- 394	8169	14133
**Needle biopsy**	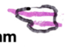 5 mm	69 µg +/- 29 µg	1843 +/- 129	1720 +/- 279	6792	5243

Supplementary figure 12: Increased phosphoproteome coverage for FFPE samples with multiplexed TMT:
a: Identified peptides and phosphopeptides in label-free, single-shot analyses of FFPE biopsies and FFPE resected tissue samples. Source data are provided as a Source Data file.
b: Summary of protein yields, proteome, and phosphoproteome coverage for different pathology sample types and LFQ and TMT proteomics methods. Protein yield for biopsy samples was calculated from samples of 3x10µm slices and only unique proteins and no isoforms were counted for quantified proteins in TMT11 experiment. Protein yield and LFQ ID numbers are shown as averages +/- standard deviation, for TMT quantification numbers the overlap between two replicate plexes is shown. Hematoxylin and eosin staining in the first column of the table show the actual sizes of examples of resected tissue and needle biopsy FFPE samples with a 5 mm bar for scale. Source data are provided as a Source Data file.

Supplementary Fig. 13

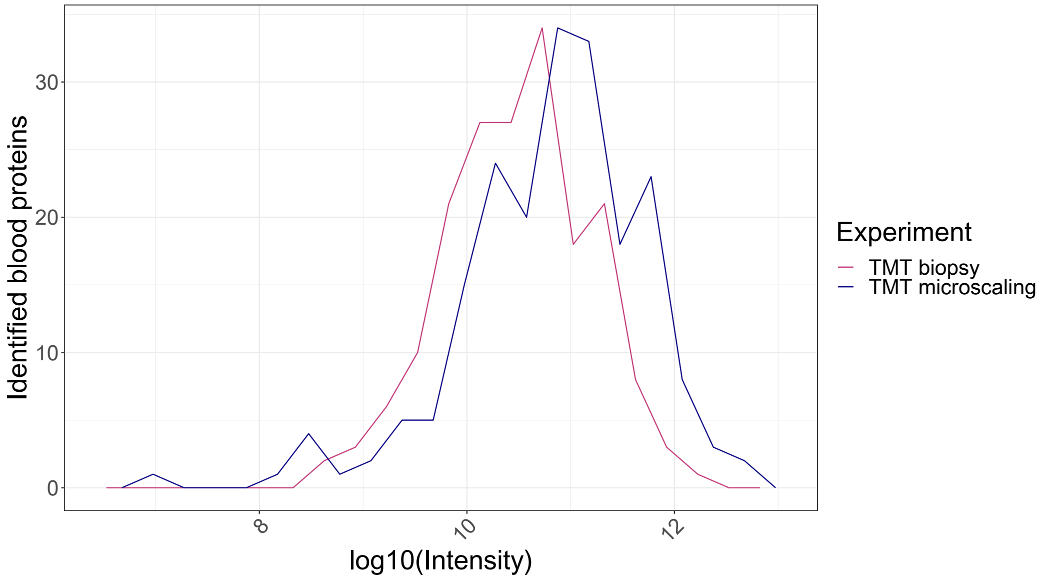

Supplementary figure 13: No increased influence of blood proteins observed in FFPE needle biopsies compared to small amounts of resected tissue.
The log 10 intensity distribution of blood proteins (from Chambers et al., 2013[2]) in microscaled TMT is shown in blue and in TMT biopsies in magenta. Source data are provided as a Source Data file.

Supplementary Table 1: Number of ADC and SCC cases per 24h or 72h fixation time used for LFQ and TMT experiments. Numbers for microscaled TMT listed in brackets.

Cancer type	Fixation time	n for LFQ experiment	n for TMT experiments
ADC	24h	10	3 (2)
ADC	72h	6	2 (2)
SCC	24h	8	3 (3)
SCC	72h	6	2 (1)

Supplementary Table 2: NSCLC-relevant marker proteins and the experiment in which they were quantified

**Protein**	**UniProt ID**	**Quantified by**	**Link to NSCLC**

NFE2L2 (NRF2)	Q16236	—-	NRF2 is a transcription factor involved in oxidative stress response [3]
KEAP1	Q14145	TMT equal, TMT micro, TMT biopsy	Mutations have been shown in lung ADC [4], inactivating mutations of KEAP1 are thought to be related to chemotherapy resistance [3]
CUL3	Q13618	LFQ, TMT equal, TMT micro	E3 ubiquitin ligase, mutations have been shown in lung SCC [5]
NQO1	P15559	TMT equal, TMT micro	A marker for NRF2 activation [6]
GSK3B	P49841	TMT equal, TMT micro	Member of PI3K signaling pathway and mediator of NRF2 degradation [6]
CDKN1A (p21)	P38936	TMT equal	Involved in KEAP1-independent, p53 regulated NRF2 activation [6,7]
SQSTM1 (p62)	Q13501	TMT equal, TMT micro	Involved in KEAP1-independent NRF2 activation [6]
DSG3	P32926	TMT equal, TMT micro	DSG3 was shown to be highly expressed in lung SCC compared to ADC [8]

SOX2	P48431	TMT equal, TMT micro	SOX2 is overexpressed in lung SCC and drives proliferation [9]
TP63	Q9H3D4	TMT equal	Immunohistochemistry (IHC) marker SCC
KRT5	P13647	TMT equal, TMT micro, TMT biopsy	IHC marker SCC
KRT6	P02538/P04259	TMT equal, TMT micro, TMT biopsy	IHC marker SCC
NKX2-1 (TTF1)	P43699	TMT equal, TMT micro, TMT biopsy	IHC marker ADC
KRT7	P08729	TMT equal, TMT micro, TMT biopsy	IHC marker ADC
NAPSA	O96009	TMT equal, TMT micro, TMT biopsy	IHC marker ADC
SFTPA1	Q8IWL2	TMT equal, TMT biopsy	IHC marker lung
EPCAM	P16422	TMT equal, TMT micro, TMT biopsy	IHC marker epithelial cells
EGFR	P00533	TMT equal, TMT micro, TMT biopsy	EGFR is a commonly mutated driver oncogene in lung ADC [4]
SMARCA2	P51531	TMT equal, TMT micro, TMT biopsy	High expression in lung cancer is associated with good prognosis [10]
AKT2	P31751	TMT equal, TMT micro, TMT biopsy	Member of the PI3K signaling pathway, overexpression has been shown in both ADC and SCC [11]

CEACAM5	P06731	TMT equal, TMT micro, TMT biopsy	Drives proliferation, overexpressed in NSCLC [12]
MTOR	P42345	TMT equal, TMT micro, TMT biopsy	A serine-threonine kinase that induces proliferation and a member of the PI3K signaling pathway [11]
KRAS	P01116	TMT equal, TMT micro, TMT biopsy	Member of RTK/RAS pathway, which is often altered in NSCLC [13]
AKT1	Q96B36	TMT equal, TMT micro, TMT biopsy	Member of the PI3K signaling pathway, sometimes mutated in lung ADC [4]
ERBB2	P04626	TMT equal, TMT micro, TMT biopsy	ERBB2 is driving lung ADC and is a target for receptor tyrosine kinase inhibitor therapy [4]
ARAF	P10398	TMT equal, TMT micro, TMT biopsy	Member of RTK/RAS pathway, which is often altered in NSCLC [13]

PTEN	P60484	TMT equal, TMT micro, TMT biopsy	Tumor suppressor, inhibits PI3K signaling pathway [11]
MAP2K1 (MEK1)	Q02750	TMT equal, TMT micro, TMT biopsy	Member of the RTK/RAS pathway, was shown to be mutated in some lung ADC cases [4,13]
MET	P08581	TMT equal, TMT micro, TMT biopsy	MET amplification was shown as a driver event in ADC [4]
BRAF	P15056	TMT equal, TMT biopsy	Mutations in the oncogene BRAF are commonly found in ADC [4]
TP53	P04637	TMT equal	Mutations in tumor suppressor gene TP53 are common in lung ADC [4]

Supplementary References

1. Krokhin, O. V. & Spicer, V. Peptide retention standards and hydrophobicity indexes in reversed-phase high-performance liquid chromatography of

peptides. *Anal. Chem.* **81**, 9522–9530 (2009).

2. Chambers, A. G., Percy, A. J., Hardie, D. B. & Borchers, C. H. Comparison of proteins in whole blood and dried blood spot samples by LC/MS/MS. *J. Am. Soc. Mass Spectrom.* **24**, 1338–1345 (2013).
3. Singh, A. *et al.* Dysfunctional KEAP1-NRF2 interaction in non-small-cell lung cancer. *PLoS Med.* **3**, 1865–1876 (2006).
4. Cancer Genome Atlas Research Network, Cancer Genome Atlas Research Network, T. C. G. A. R., Network, T. C. G. A. R. & Cancer Genome Atlas Research Network, T. C. G. A. R. Comprehensive molecular profiling of lung adenocarcinoma. *Nature* **511**, 543–550 (2014).
5. Network, T. C. G. A. R. Comprehensive genomic characterization of squamous cell lung cancers. *Nature* **489**, 519–525 (2012).
6. Best, S. A. & Sutherland, K. D. "Keaping" a lid on lung cancer: the Keap1-Nrf2 pathway. *Cell Cycle* vol. 17 1696–1707 (2018).
7. Chen, W. *et al.* Direct Interaction between Nrf2 and p21Cip1/WAF1 Upregulates the Nrf2-Mediated Antioxidant Response. *Mol. Cell* **34**, 663–673 (2009).
8. Savci-Heijink, C. D. *et al.* The role of desmoglein-3 in the diagnosis of squamous cell carcinoma of the lung. *Am. J. Pathol.* **174**, 1629–1637 (2009).
9. Mollaoglu, G. *et al.* The Lineage-Defining Transcription Factors SOX2 and NKX2-1 Determine Lung Cancer Cell Fate and Shape the Tumor Immune Microenvironment. *Immunity* **49**, 764-779.e9 (2018).
10. Guerrero-Martínez, J. A. & Reyes, J. C. High expression of SMARCA4 or SMARCA2 is frequently associated with an opposite prognosis in cancer. *Sci. Rep.* **8**, 1–17 (2018).
11. Fumarola, C., Bonelli, M. A., Petronini, P. G. & Alfieri, R. R. Targeting

PI3K/AKT/mTOR pathway in non small cell lung cancer. *Biochemical Pharmacology* vol. 90 197–207 (2014).

12. Zhang, X., Han, X., Zuo, P., Zhang, X. & Xu, H. CEACAM5 stimulates the progression of non-small-cell lung cancer by promoting cell proliferation and migration. *J. Int. Med. Res.* **48**, (2020).

13. Sanchez-Vega, F. *et al.* Oncogenic Signaling Pathways in The Cancer Genome Atlas. *Cell* **173**, 321-337.e10 (2018).
