# Supplementary material for: Comprehensive micro-scaled proteome and phosphoproteome characterization of archived retrospective cancer repositories
Source: Nat Commun. 2021 Jun 11;12:3576. doi: 10.1038/s41467-021-23855-w (PMC8196151; doi:10.1038/s41467-021-23855-w)
Supplement: Supplementary file 3 — Description of Additional Supplementary Files [file 41467_2021_23855_MOESM3_ESM.docx]

**Description of Additional Supplementary Files**

**File Name:** Supplementary Data 1

**Description:** Protein intensities for comparison of DTR, SDC, and SDS-SP3 protocols.

**File Name:** Supplementary Data 2

**Description:** Data for LFQ comparison of 30 NSCLC cases.

**File Name:** Supplementary Data 3

**Description:** Global proteome data for equal loading TMT of 10 NSCLC cases.

**File Name:** Supplementary Data 4

**Description:** Phosphoproteome data for equal loading TMT of 10 NSCLC cases.

**File Name:** Supplementary Data 5

**Description:** Global proteome data for microscaled TMT of 8 NSCLC cases.

**File Name:** Supplementary Data 6

**Description:** Phosphoproteome data for microscaled TMT of 8 NSCLC cases.

**File Name:** Supplementary Data 7

**Description:** Summed reporter ion intensities for microscaled and biopsy TMT.

**File Name:** Supplementary Data 8

**Description:** Summed phosphopeptide reporter ion intensities for microscaled and biopsy TMT.
