# Supplementary material for: Comprehensive micro-scaled proteome and phosphoproteome characterization of archived retrospective cancer repositories
Source: Nat Commun. 2021 Jun 11;12:3576. doi: 10.1038/s41467-021-23855-w (PMC8196151; doi:10.1038/s41467-021-23855-w)
Supplement: Supplementary file 12 — Reporting Summary [file 41467_2021_23855_MOESM12_ESM.pdf]

nature research

Corresponding author(s): Philipp Mertins, Frederick Klauschen

Last updated by author(s): May 3, 2021

Reporting Summary

Nature Research wishes to improve the reproducibility of the work that we publish. This form provides structure for consistency and transparency in reporting. For further information on Nature Research policies, see our Editorial Policies and the Editorial Policy Checklist.

Statistics

For all statistical analyses, confirm that the following items are present in the figure legend, table legend, main text, or Methods section.

n/a	Confirmed	
☐	☒	The exact sample size (n) for each experimental group/condition, given as a discrete number and unit of measurement
☐	☒	A statement on whether measurements were taken from distinct samples or whether the same sample was measured repeatedly
☐	☒	The statistical test(s) used AND whether they are one- or two-sided<br>*Only common tests should be described solely by name; describe more complex techniques in the Methods section.*
☐	☒	A description of all covariates tested
☐	☒	A description of any assumptions or corrections, such as tests of normality and adjustment for multiple comparisons
☐	☒	A full description of the statistical parameters including central tendency (e.g. means) or other basic estimates (e.g. regression coefficient) AND variation (e.g. standard deviation) or associated estimates of uncertainty (e.g. confidence intervals)
☐	☒	For null hypothesis testing, the test statistic (e.g. F, t, r) with confidence intervals, effect sizes, degrees of freedom and P value noted<br>*Give P values as exact values whenever suitable.*
☒	☐	For Bayesian analysis, information on the choice of priors and Markov chain Monte Carlo settings
☒	☐	For hierarchical and complex designs, identification of the appropriate level for tests and full reporting of outcomes
☐	☒	Estimates of effect sizes (e.g. Cohen's d, Pearson's r), indicating how they were calculated

Our web collection on statistics for biologists contains articles on many of the points above.

Software and code

Policy information about availability of computer code

Data collection	Raw data was collected on a QExactive Plus mass spectrometer with Thermo Xcalibur software version 3.0.63 (August 2013) and QExactive HF-X mass spectrometer with Thermo Xcalibur software version 4.1.31.9 (June 2017)
Data analysis	Proteome database searches were performed using MaxQuant (v1.6.3.3). Data analysis was performed with R version 3.6.0 using the limma R package (V3.44.3), corrplot R package (V0.84), clusterProfiler R package (V 3.16.1) and ssGSEA was performed using ssGSEA and PTM-SEA tools.

For manuscripts utilizing custom algorithms or software that are central to the research but not yet described in published literature, software must be made available to editors and reviewers. We strongly encourage code deposition in a community repository (e.g. GitHub). See the Nature Research guidelines for submitting code & software for further information.

Data

Policy information about availability of data

All manuscripts must include a data availability statement. This statement should provide the following information, where applicable:

- Accession codes, unique identifiers, or web links for publicly available datasets
- A list of figures that have associated raw data
- A description of any restrictions on data availability

Raw data files and MaxQuant output files are available via the PRIDE repository (accesion number PXD024800).
The human reference proteome (UP000005640, downloaded 01/2019) was used in MaxQuant.

Field-specific reporting

Please select the one below that is the best fit for your research. If you are not sure, read the appropriate sections before making your selection.

- [x] Life sciences
- [ ] Behavioural & social sciences
- [ ] Ecological, evolutionary & environmental sciences

For a reference copy of the document with all sections, see nature.com/documents/nr-reporting-summary-flat.pdf

Life sciences study design

All studies must disclose on these points even when the disclosure is negative.

Sample size	Sample size was chosen based on available channels in multiplexed isotope labeling at the beginning of the study.
Data exclusions	For the heat incubation experiment, HEK293 cells were embedded into a paraffin block, but results were not shown because of issues during the embedding process. Results for two replicates of the label-free single-shot phosphopeptide enrichment from FFPE resected tissues were not shown because of issues with the pipetting robot.
Replication	Cell line experiments were performed in four replicates, TMT experiments were analyzed in duplicates (samples randomly distributed into different TMT channels in each replicate), LFQ experiments were measured in duplicates. All replicates were successful.
Randomization	Samples were chosen for the TMT subsets based on available material and TMT samples were randomly assigned to different channels in each replicate
Blinding	Patient samples were assigned anonymous IDs and randomly divided into two batches for sample processing. For cell culture experiments, fresh-frozen and formalin-fixed cells were alternated for TMT channels to minimize interference.

Reporting for specific materials, systems and methods

We require information from authors about some types of materials, experimental systems and methods used in many studies. Here, indicate whether each material, system or method listed is relevant to your study. If you are not sure if a list item applies to your research, read the appropriate section before selecting a response.

Materials & experimental systems

n/a	Involved in the study	
[ ]	[x]	Antibodies
[ ]	[x]	Eukaryotic cell lines
[x]	[ ]	Palaeontology and archaeology
[x]	[ ]	Animals and other organisms
[ ]	[x]	Human research participants
[x]	[ ]	Clinical data
[x]	[ ]	Dual use research of concern

Methods

n/a	Involved in the study	
[x]	[ ]	ChIP-seq
[x]	[ ]	Flow cytometry
[x]	[ ]	MRI-based neuroimaging

Antibodies

Antibodies used	CK5/6, clone EP24, EP67, abcam in 1:100<br>CK7, clone OV-TL12/30, Dako in 1:1000
Validation	The antibody stainings were done in the routine lab of the pathology institute according to established and accredited protocols.

Eukaryotic cell lines

Policy information about cell lines

Cell line source(s)	HEK293 cells were grown from lab stock that had been purchased from ATCC.
Authentication	Cell lines were not authenticated because it was not relevant for this experiment
Mycoplasma contamination	Cells were originally tested to not contain mycoplasma contamination.
Commonly misidentified lines (See ICLAC register)	No commonly misidentified cell lines were used in this study.

Human research participants

Policy information about studies involving human research participants

Population characteristics	30 NSCLC cases, 16 ADC, 14 SCC. 14 out of 30 patients were female, 16 male. 18 FFPE samples had been fixed 24h, 12 had been formalin fixed for 72h
Recruitment	Samples were retrospectively collected from archived FFPE material that had been acquired in the Charité pathology department.
Ethics oversight	Informed consent was obtained from all patients through the patient contract in accordance with institutional guidelines approved by the ethics board at the Charité Universitätsmedizin Berlin.

Note that full information on the approval of the study protocol must also be provided in the manuscript.
